# MOLEonline: a web-based tool for analysing channels, tunnels, and pores (2025 update)

**DOI:** 10.1093/bioinformatics/btaf486

**Published:** 2025-09-03

**Authors:** Tomáš Raček, Dušan Vel’ký, Gabriela Bučeková, Ondřej Schindler, Ivana Hutařová Vařeková, Anna Špačková, Václav Bazgier, Karel Berka, Radka Svobodová

**Affiliations:** National Centre for Biomolecular Research, Faculty of Science, Masaryk University, Kamenice 5, 625 00 Brno, Czech Republic; CEITEC—Central European Institute of Technology, Masaryk University, Kamenice 5, 625 00 Brno, Czech Republic; CEITEC—Central European Institute of Technology, Masaryk University, Kamenice 5, 625 00 Brno, Czech Republic; National Centre for Biomolecular Research, Faculty of Science, Masaryk University, Kamenice 5, 625 00 Brno, Czech Republic; CEITEC—Central European Institute of Technology, Masaryk University, Kamenice 5, 625 00 Brno, Czech Republic; National Centre for Biomolecular Research, Faculty of Science, Masaryk University, Kamenice 5, 625 00 Brno, Czech Republic; CEITEC—Central European Institute of Technology, Masaryk University, Kamenice 5, 625 00 Brno, Czech Republic; CEITEC—Central European Institute of Technology, Masaryk University, Kamenice 5, 625 00 Brno, Czech Republic; Department of Physical Chemistry, Faculty of Science, Palacký University, 17. listopadu 1192/12, 771 46 Olomouc, Czech Republic; Department of Physical Chemistry, Faculty of Science, Palacký University, 17. listopadu 1192/12, 771 46 Olomouc, Czech Republic; Department of Physical Chemistry, Faculty of Science, Palacký University, 17. listopadu 1192/12, 771 46 Olomouc, Czech Republic; Department of Physical Chemistry, Faculty of Science, Palacký University, 17. listopadu 1192/12, 771 46 Olomouc, Czech Republic; National Centre for Biomolecular Research, Faculty of Science, Masaryk University, Kamenice 5, 625 00 Brno, Czech Republic; CEITEC—Central European Institute of Technology, Masaryk University, Kamenice 5, 625 00 Brno, Czech Republic

## Abstract

**Summary:**

MOLEonline is an interactive, web-based tool designed to detect and analyse channels (pores and tunnels) within protein structures. The latest version of MOLEonline addresses the limitations of its predecessor by integrating the Mol* viewer for visualization and offering a streamlined, fully interactive user experience. The new features include colouring tunnels in the 3D viewer based on their physicochemical properties. A 2D representation of the protein structure and calculated tunnels is generated using 2DProts. Users can now store tunnels directly in the mmCIF file format, facilitating sharing via the community-standard FAIR format for structural data. In addition, the ability to store and load computation settings ensures the reproducibility of tunnel computation results. Integration with the ChannelsDB 2.0 database allows users to access precomputed tunnels.

**Availability and implementation:**

The MOLEonline application is freely available at https://moleonline.cz with no login requirement, its source code is stored at GitHub under the MIT licence at https://github.com/sb-ncbr/moleonline-web, and archived at Figshare at https://doi.org/10.6084/m9.figshare.29816174.

## Graphical Abstract

**Figure:**
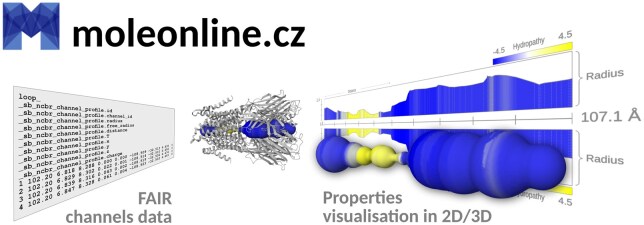

## 1 Introduction

In protein structures, active sites can be either on the surface or buried within the structure. Buried active sites are connected to outer space through tunnels or pores. A tunnel leads exclusively to the active site, whereas a pore extends through the entire protein structure. These tunnels and pores often have functional roles, which typically involve transporting substances between different spatial regions (Marques *et al.* 2017). Over 64% of enzymes possess buried active sites, which are linked to the external environment by tunnels, facilitating the transport of substrates and products (Pravda *et al.* 2014). Radius and other physicochemical properties can affect which molecule can lead through the tunnel and consequently react with the buried active site and play a crucial role in enzyme activity, specificity, and stability (Kokkonen *et al.* 2019). Pores in proteins, such as transmembrane pores, play a crucial role in cellular function by facilitating the transport of ions, molecules, and other substances across biological membranes. They perform various functions, including selective transport, rectification (one-way transport), and gating (opening and closing) (Ayub and Bayley 2016). For example, ion channels are essential for nerve signalling (Hille 2022), muscle contraction (Perez-Zoghbi *et al.* 2009), and maintaining cellular electrical balance. Additionally, transmembrane pores transport nutrients, waste products, and signalling molecules, which are vital for maintaining cellular health and function and ensuring transport selectivity. The significance of tunnels, channels, and pores in biomacromolecular structures highlights the need for tools to detect and analyse these features. Several software tools have been developed, offering varying levels of functionality and accessibility. (Pravda *et al.* 2018, Stourac *et al.* 2019, Vavra *et al.* 2019) A version of MOLE for command-line computing can be used for larger datasets (Sehnal *et al.* 2013). Among these solutions, web-based tools are highly valued by structural biologists for their accessibility across various devices, platforms, and operating systems, as well as their ability to integrate with other bioinformatics tools and pipelines.

## 2 Description of the tool

The algorithm for tunnel and pore detection in MOLEonline is based on our previously published approach (Pravda *et al.* 2018). The tool includes new features and an improved web interface. The main window features a 3D visualization of the protein in the Mol* viewer (Sehnal *et al.* 2021) [instead of older LiteMol (Sehnal *et al.* 2017)], in which users can colour the tunnel according to the values of the selected physicochemical property along the tunnel profile. Additionally, structures can be displayed in 2DProts (Hutařová Vařeková *et al.* 2021), allowing tunnels and the structure to be visualized in a 2D projection. Newly in the top left corner, two main tabs are available: *Compute*, which allows users to perform new tunnel and pore calculations and modify the parameters of those computations; and *Channels*, which contains results of the computations and precomputed tunnels and pores loaded from the ChannelsDB 2.0 database (Špačková *et al.* 2024).

### 2.1 User workflow

This new version of MOLEonline builds upon the previous version, offering several improvements to enhance user experience, separating the computation settings and analysis of results. Initially, an input structure has to be uploaded in the mmCIF or older PDB format or downloaded directly from the PDBe database by entering its PDB ID code. MOLEonline also offers the option to select a biologically active unit based on PDBe annotations.

**Figure 1.**
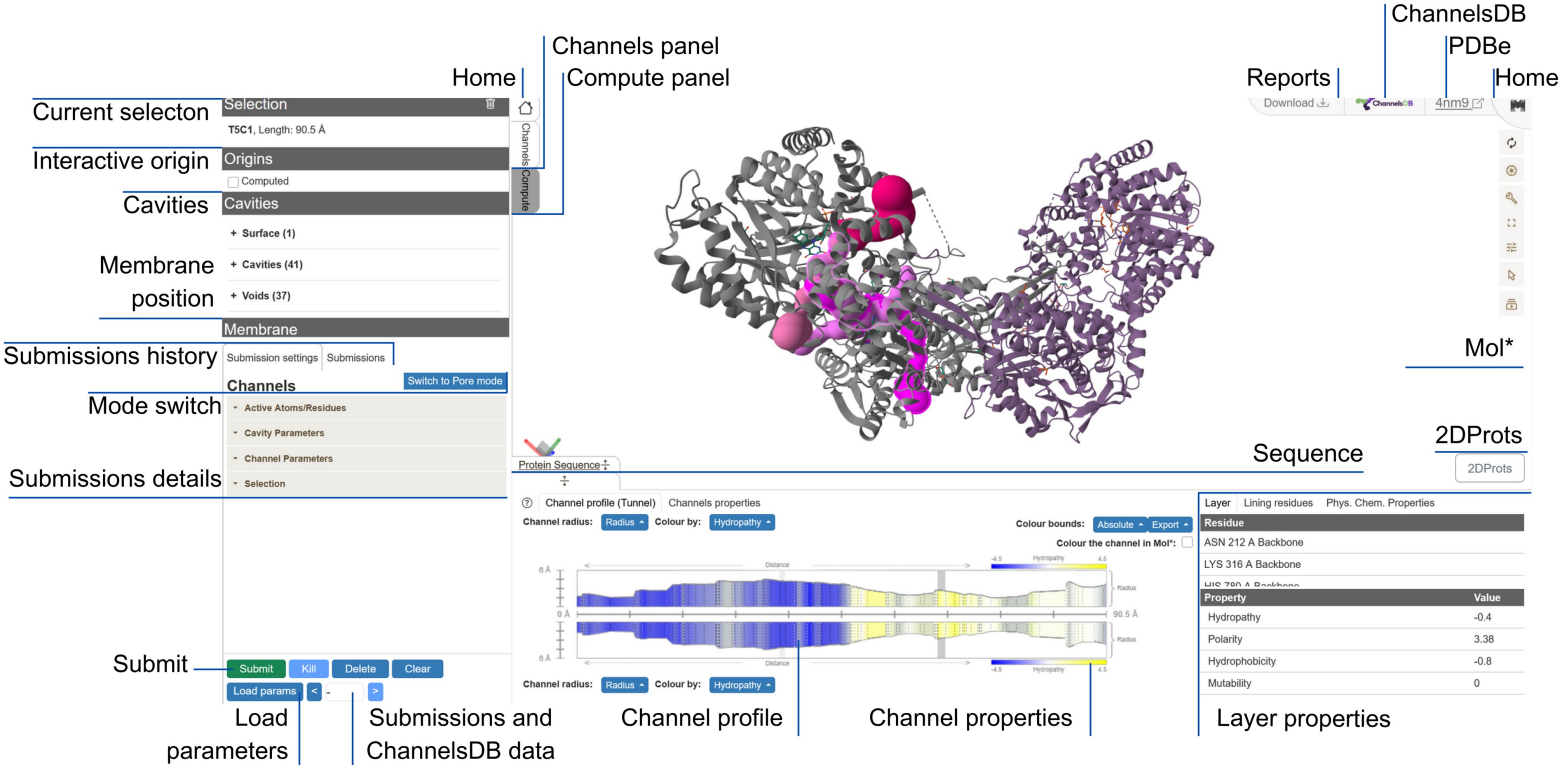
Snapshot of the main user interface loaded with a protein structure (PDB ID: *4nm9*) with channels loaded from ChannelsDB 2.0. The interface enables the computation of channels, their interactive 1D visualization and 3D visualization in Mol*.

MOLEonline has a redesigned user interface described in Fig. 1 providing two modes of calculation: *Channels mode* (default), designed to identify tunnels and merged pores, and *Pore mode*, tailored for characterizing transmembrane pores. The main workflow is controlled via two panels. In *Compute panel*, the user can adjust the settings and run the calculation using the *Submit* button. After the calculation, the list of discovered tunnels is shown in *Channels panel*. A detailed description of the calculation options using the modes mentioned above can be found at https://github.com/sb-ncbr/moleonline-web/wiki. The most notable new features are described here in the following sections.

### 2.2 Colouring the channels in Mol*

Based on the selection of the tunnel’s physicochemical properties (such as polarity, charge, and other relevant factors), tunnel visualization can be enhanced by applying colour coding. This approach allows for a more intuitive and detailed understanding of the tunnel’s surroundings. By assigning different colours to various properties, one can quickly identify regions with specific characteristics, facilitating more effective analysis and interpretation of the tunnel environment. See *Example I* for a particular case.

### 2.3 Connection to ChannelsDB 2.0

The latest results from MOLE and CAVER (Chovancova *et al.* 2012), now integrated into the ChannelsDB 2.0 (Špačková *et al.* 2024) database, are available through the updated MOLEonline platform (2025 version). These results encompass a variety of channels, including those manually curated from the literature, computationally derived from catalytic sites listed in the Catalytic Site Atlas [CSA (Furnham *et al.* 2014)], associated with cofactors, containing cognate ligands (Vavra *et al.* 2024), and computed from AlphaFill (Hekkelman *et al.* 2023) structures. Additionally, the database includes transmembrane pores.

### 2.4 2DProts integration

2DProts (Hutařová Vařeková *et al.* 2021) is a software tool designed for the visualization of protein secondary structure through 2D diagrams. It forms part of the CATH protein family database (Sillitoe *et al.* 2021). We integrated 2DProts into MOLEonline, enabling users to view 2D representations of protein structures alongside their associated channels (see an example in Fig. 3b). This integration provides an intuitive overview of the channel system within the protein structure.

### 2.5 Towards FAIRification of channel data

FAIR data are essential for modern science, ensuring that research findings can be reliably interpreted and reproduced. If conclusions based on the MOLEonline analyses are shared in a research paper without providing the underlying data, reproducing the results can be challenging. To support interoperability and reusability, MOLEonline now allows channel data to be stored in the standard mmCIF format, making it easier to exchange with compatible applications, e.g. Mol* viewer. Newly added categories conform to the definitions in the mmCIF extension dictionary, which is referenced in the Wiki. Additionally, computation settings can be saved in a JSON format and subsequently reloaded, allowing users to replicate analyses with consistent parameters and ensuring reliable channel detection and characterization. Note that all results are available under the CC BY 4.0 licence.

### 2.6 Limitations

Despite improvements in the latest version, MOLEonline has certain inherent limitations. Calculations are performed by an API server that processes tasks sequentially, meaning users may experience longer wait times under high load. For a single computation, proteins with up to 60 000 residues are manageable. The 3D visualization runs in the client’s browser, so channel colouring may take longer on older hardware. Pore calculations using the Memembed software (Nugent and Jones 2013) for membrane orientation prediction are not deterministic, potentially yielding slightly different results across multiple runs. Finally, MOLEonline cannot generate mmCIF data if the input is in the deprecated PDB format.

## 3 Results and discussion

We extensively tested the application to confirm that it is stable and reliable. Furthermore, we provide two use cases that demonstrate various applications of MOLEonline.

### 3.1 Example I: Pentameric ligand-gated ion channels

**Figure 2.**
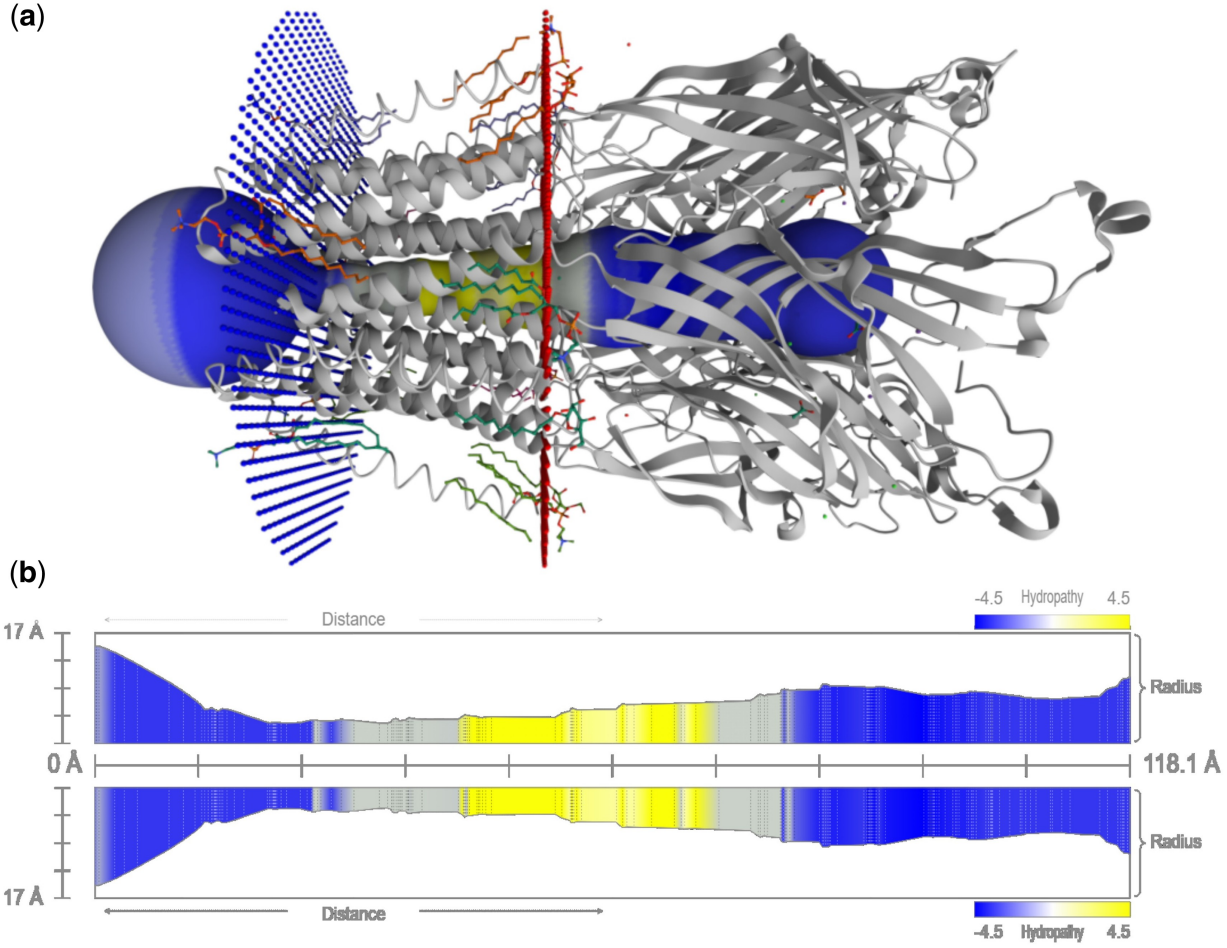
Pore visualization in MOLEonline (PDB ID: *6hzw*). (a) The pore is coloured based on hydropathy properties along its length. (b) A 2D map displays the pore’s radius, coloured according to hydropathy, and divided into layers based on the surrounding amino acids.

The pentameric ligand-gated ion channels (pLGICs) from *Gloeobacter violaceus* (GLIC) advanced our understanding of the pLGICs family. These channels are essential for quick transmission of nerve signals in both the central and peripheral nervous system. Dysfunction of pLGICs is associated with severe neurological diseases and conditions. GLIC activation is pH-dependent, and its gating mechanism involves loop F rather than the classic orthosteric site (Sauguet *et al.* 2015, Hu *et al.* 2018). Using MOLEonline, the transmembrane pore of the GLIC pentameric ligand-gated ion channel (PDB entry *6hzw*) was analysed. The calculated pore is 118 Å long, with a bottleneck radius of 3.1 Å (as shown in Fig. 2), allowing the transport of various molecules. The pore is visualized with colour gradients representing hydropathy along its structure.

### 3.2 Example II: Cytochrome P450 2C8

**Figure 3.**
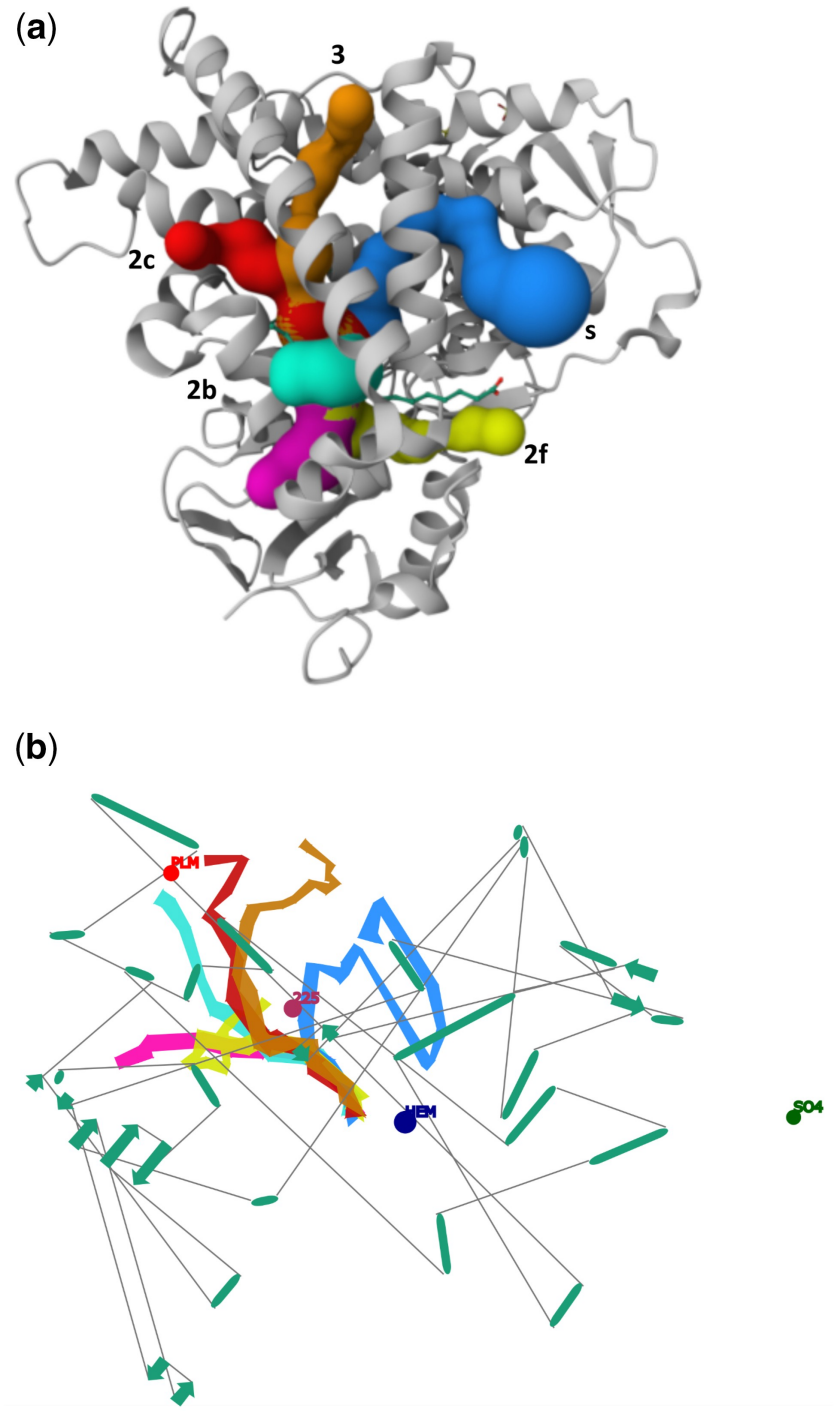
Cytochrome P450 2C8 (PDB ID: *2nnj*). (a) Annotation of protein channels defined in relation to the annotated SSEs. (b) Visualization of the protein with tunnels analysed using 2DProts, outlying individual secondary structures along the identified channels.

Cytochrome P450 2C8 (PDB entry *2nnj*) is a major drug-metabolising enzyme in the human liver, playing a key role in the oxidative metabolism of at least 5% of clinical drugs. It is the predominant hepatic P450 responsible for the 6α-hydroxylation of paclitaxel and the epoxidation of arachidonic acid (Schoch *et al.* 2008). Cytochrome protein secondary structure elements (SSEs) are valuable tools for navigation within protein structures. Specific SSE annotations in protein families allow for consistent naming of equivalent SSEs in homologous proteins, facilitating detailed analysis of these groups for a comprehensive family overview and improved study of individual proteins and their channels (Midlik *et al.* 2021). Using SSEs naming, MOLEonline identified six tunnels (Fig. 3) as described in the literature and assigned the following nomenclature: Solvent channel, Channel 2b, 2ac, 2f, and 3. The lengths of these tunnels range from 31.8 Å for Channel 2b to 50.8 Å for Channel 3. All tunnels lead to the active site, which is HEM. Channel 2b has the widest bottleneck radius of 2.1 Å, while Channel 3 has the narrowest, measuring 1.1 Å.

## 4 Conclusion

MOLEonline (version 2025) enables interactive, platform-independent analysis of channels, tunnels, and pores, visualized in Mol*. It detects and analyses structural and physicochemical properties, including transmembrane pores. Integrated with ChannelsDB 2.0, it helps identify biological functions by comparing detected features with annotated structures. It also incorporates 2DProts for generating 2D representations and supports 3D property visualization along tunnel profiles. Finally, this version also addresses the need for FAIR data representations, allowing the tunnel data to be stored in the standard mmCIF format.
